# Identifying Lipid Metabolism‐Related Therapeutic Targets and Diagnostic Markers for Lung Adenocarcinoma by Mendelian Randomization and Machine Learning Analysis

**DOI:** 10.1111/1759-7714.70020

**Published:** 2025-03-19

**Authors:** Su Wei, Zhou Guangyao, Tian Xiangdong, Guo Feng, Zhang Lianmin, Zhang Zhenfa

**Affiliations:** ^1^ Department of Endoscopy Tianjin Medical University Cancer Institute and Hospital, National Clinical Research Center for Cancer Tianjin China; ^2^ Key Laboratory of Cancer Prevention and Therapy Tianjin China; ^3^ Tianjin's Clinical Research Center for Cancer Tianjin China; ^4^ Department of Lung Cancer Tianjin Medical University Cancer Institute and Hospital, National Clinical Research Center of Cancer Tianjin China

**Keywords:** lipid metabolism, lung adenocarcinoma, machine learning, Mendelian randomization

## Abstract

**Background:**

Lipid metabolic disorders are emerging as a recognized influencing factors of lung adenocarcinoma (LUAD). This study aims to investigate the influence of lipid metabolism‐related genes (LMRGs) on the diagnosis and treatment of LUAD and to identify significant biomarkers.

**Methods:**

DESeq2 and robust rank aggregation (RRA) analyses were employed to determine the differential expression of LMRGs from TCGA‐LUAD and five GEO datasets. Mendelian randomization (MR) was conducted utilizing protein quantitative trait loci (pQTLs) in the deCODE, prot‐a, and UKB‐PPP Study to estimate causal relationships between plasma proteins and LUAD within the ieu‐a‐984, ieu‐a‐965, and FinnGen R10 cohorts as potential drug targets of LUAD. Subsequently, an optimal machine learning model for diagnosing LUAD was established by comparing four models: support vector machine, random forest (RF), glmBoost, and eXtreme Gradient Boosting. Finally, the diagnostic performance of five plasma proteins was validated through nomogram analysis, calibration curve assessment, decision curve analysis (DCA), independent internal and external datasets.

**Result:**

A total of five biomarkers were identified from 1034 LMRGs via MR and differential expression analysis. TNFRSF21 exhibited a positive association with LUAD risk; conversely, BCHE, FABP4, LPL, and PLBD1 demonstrated negative correlations with this risk. The RF machine learning model was determined to be the optimal model for diagnosing LUAD using these five plasma proteins. Ultimately, nomogram construction, calibration curve analysis, DCA, as well as independent internal and external dataset validation confirmed that these biomarkers exhibit excellent diagnostic performance.

**Conclusions:**

BCHE, FABP4, LPL, PLBD1, and TNFRSF21 represent potential novel reliable diagnostic markers as well as therapeutic targets for LUAD.

## Graphical abstract

This study aims to investigate the influence of lipid metabolism‐related genes (LMRGs) on the diagnosis and treatment of lung adenocarcinoma (LUAD) and to identify significant biomarkers. Through Mendelian randomization and machine learning, we identified five biomarkers associated with LMRGs as potential diagnostic markers and therapeutic targets for LUAD. The results of our study have excellent performance in internal and external validation databases.

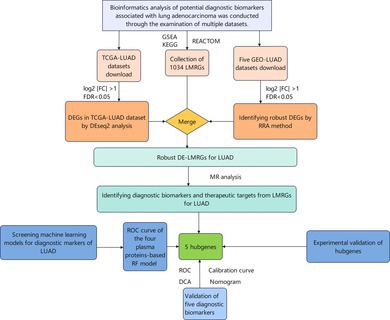

## 1 Introduction

Lung cancer is a highly prevalent and fatal malignant tumor worldwide [1], accounting for approximately 20% of global cancer‐related deaths [2, 3]. Among the primary lung cancers, lung adenocarcinoma (LUAD) represents the most common histological subtype [4]. The advent of multislice spiral CT technology and the widespread utilization of low‐dose spiral CT screening have led to an increased detection rate of LUAD. However, these methods suffer from limited specificity and false‐positive results [5]. Considering the limitations of existing screening methods, there is an urgent need for more precise, efficient, and convenient screening approaches to further improve diagnostic efficiency. Consequently, it becomes imperative to identify reliable diagnostic marker genes.

Lipid metabolism is closely associated with the occurrence and progression of cancer [6, 7]. Tumor cells exhibit enhanced proliferative capacity and require a greater energy supply compared to normal cells [8, 9]. There is evidence that suggests a correlation between abnormal lipid metabolism and various cancers, including breast cancer, postmenopausal tumors, colorectal cancer, endometrial cancer, pancreatic cancer, and biliary tract cancer [10, 11, 12]. Lipid metabolism‐related genes (LMRGs) have potential as biomarkers for tumor diagnosis since dysregulation of lipid metabolism plays a significant role in the pathogenesis and progression of malignancies [13, 14, 15, 16]. Investigating the underlying mechanisms of lipid metabolism in LUAD has the potential to enhance therapeutic strategies and improve patient survival rates. Abnormal lipid metabolism is linked to lung cancer development and progression [17]. However, the role of LMRGs in LUAD remains poorly understood. Further research is required to comprehend the diagnostic and treatment value of these genes in LUAD.

Mendelian randomization (MR) serves as a valuable tool for investigating causal relationships between exposures and outcomes by leveraging genetic variation as an instrumental variable (IV) [18, 19]. By utilizing genetic differences as substitutes for exposure, MR compares health outcomes among individuals with different genetic variations to estimate the causal effects of risk factors on health while minimizing bias caused by other factors [20, 21, 22]. This approach offers a novel way to explore the causal link between illnesses and environmental variables, thereby enhancing our understanding of disease pathophysiology [23, 24, 25].

Machine learning represents a pivotal artificial intelligence technique extensively employed for the prediction of cancer diagnostic markers and holds significant promise for forecasting cancer prognosis [26, 27]. However, the development of reliable cancer outcome prediction models for routine clinical use remains a formidable challenge. In this study, we identified differentially expressed lipid metabolism‐related genes (DE‐LMRGs) in LUAD patients from the TCGA and GEO databases. Subsequently, we performed MR analysis on these DE‐LMRGs to identify five genes associated with LUAD. Finally, through comparative analysis of various machine learning methods, we validated BCHE, FABP4, LPL, PLBD1, and TNFRSF21 as potential therapeutic targets and diagnostic markers for LUAD.

## 2 Materials and Methods

### 2.1 LMGRs Availability

**FIGURE 1:**
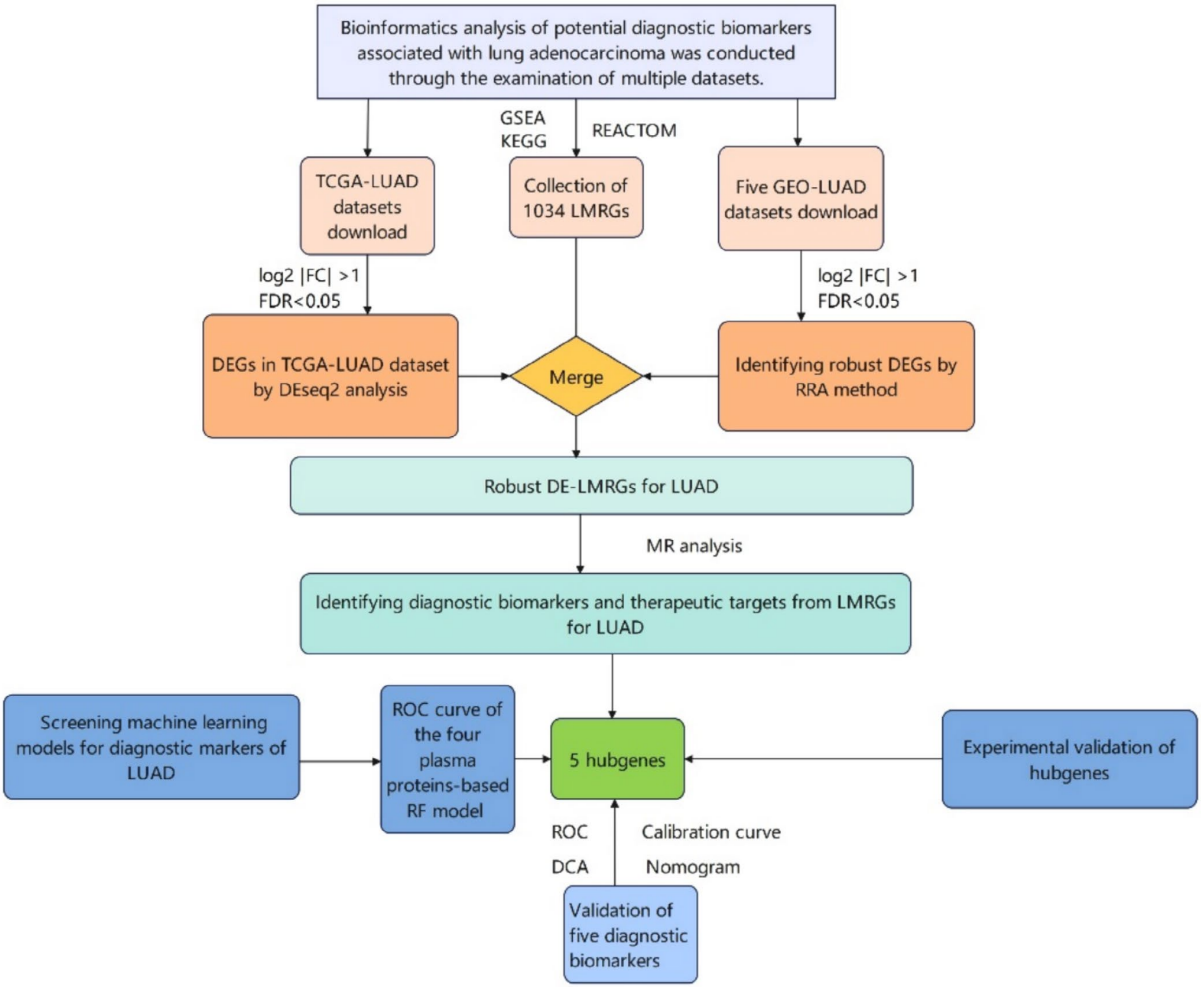
Study flow chart.

LMRGs were selected through the Gene Set Enrichment Analysis (GSEA) database, Kyoto Encyclopedia of Genes and Genomes (KEGG) database, and the REACTOME database [28]. A total of 1034 LMRGs were extracted from 1 fatty acid metabolism hallmark gene set from the GSEA database, 12 LMRG sets were extracted from the KEGG database, and 22 LMRG sets from the REACTOME database (Table S1‐1) [28]. The scheme of this study is illustrated in Figure 1.

### 2.2 Data Collection and Processing

We retrieved a total of 541 cases of LUAD and 59 normal cases from The Cancer Genome Atlas (TCGA) database (https://portal.gdc.cancer.gov/) for subsequent differential analysis. Additionally, we obtained six LUAD cohorts (GSE30219, GSE31210, GSE32863, GSE43458, GSE75037, and GSE40419) from the GEO database (https://www.ncbi.nlm.nih.gov/geo/). Among the five cohorts (GSE30219, GSE31210, GSE32863, GSE43458, and GSE75037), there were 532 LUAD tissues and 205 normal cases used to perform robust rank aggregation (RRA) analysis and receiver operating characteristic (ROC) analysis for diagnostic biomarkers. The cohort GSE40419, consisting of 87 LUAD tissues and 77 normal cases, served as an external validation cohort for diagnostic markers of LUAD. Corresponding clinical information and platform‐probe annotations were also downloaded. Detailed information about these datasets can be found in Table S2.

The LUAD GWAS data were obtained from three studies, namely FinnGen R10, TRICL, and ILCCO [29]. The FinnGen R10 study provided 1590 cases of LUAD along with 314,193 controls (https://r10.finngen.fi/). The TRICL (ieu‐a‐984) study comprised a total of 11,245 cases of LUAD and 54,619 controls (https://gwas.mrcieu.ac.uk/datasets/). Additionally, the ILCCO (ieu‐a‐965) study included 3442 cases of LUAD and 14,894 controls (https://gwas.mrcieu.ac.uk/datasets/).

### 2.3 Differential Expression Genes (DEGs) Analysis for LMRGs

The counts format of gene expression data was extracted by setting class “TCGA‐LUAD” and gene type “protein coding” using “TCGAbiolinks” package. Firstly, the TGCA‐LUAD dataset were executed DEGs analysis by DEseq2 package. Subsequently, the LMRGs were extracted from TGCA‐LUAD dataset to executed DEGs analysis. The R program “ggplot2” and “ComplexHeatmap” were used to display volcano plots and heatmap of DEGs. The cutoff values were adjusted *p* value < 0.05 and |log2 FC| > 1.

### 2.4 RRA Analysis for LMRGs

We utilized the RRA approach to integrate the aforementioned five microarray datasets, aiming to mitigate bias and errors. Initially, the “limma” package was utilized in each dataset to categorize upregulated and downregulated genes. Subsequently, robust DEGs were filtered using the “RobustRankAggreg” package on these categorized genes. A heatmap displaying the top 30 robust DEGs was generated using the “pheatmap” package. The cutoff criteria for selection were |log2FC| > 1 and FDR < 0.05.

### 2.5 MR Analysis

We selected single nucleotide polymorphisms (SNPs) that were strongly associated with plasma proteins at a significance level of *p* < 5e‐08 as IVs. These SNPs should exhibit low linkage disequilibrium (*R* ^2^ < 0.001) and are located within a genetic distance of less than 10,000 kb. SNPs with weak associations or low phenotypic variance (*F*‐test value < 10) were excluded from the analysis. The outcome data used in this study were obtained from the GWAS summary dataset available at IEU and Finngen R10 database. Specifically, we utilized the GWAS IDs ieu‐a‐984, ieu‐a‐965, and finngen_R10_C3_NSCLC_ADENO_EXALLC for our analyses. After removing SNPs with *p* < 5 × 10^5^ and plasma proteins without IVs in the LUAD dataset, we employed five different methods (MR Egger, Wald ratio, weighted median, weighted mode methods, and inverse variance weighted [IVW]) using the “TwoSampleMR” package to determine causal relationships between exposure factors and outcomes. The *p* value was used with a significant threshold of < 0.05. All five methods yielded beta values that were either greater than or less than zero, indicating positive results. Heterogeneity was assessed using Cochran's *Q* test with a significance threshold of *p* > 0.05, while pleiotropy was evaluated using the Egger intercept test, also with a significance threshold of *p* > 0.05.

### 2.6 Acquisition of Intersection Genes

Using the “VennDiagram” package, we identified 1 gene that intersected between a total of 36 genes with OR > 1 and 16 upregulated LMRGs. Additionally, by analyzing another set of 38 genes with OR < 1 and 36 downregulated LMRGs using the same package, we found a total of 4 intersected genes.

### 2.7 Constructing Machine Learning Models for LUAD

The “caret” package was utilized for the development of machine learning methods, including random forest (RF), support vector machine (SVM), generalized linear model (GLM), and eXtreme Gradient Boosting (XGB). These four machine learning methods were employed to construct prediction models with default parameters using the identified five proteins. A total of 737 LUAD samples from GSE30219, GSE31210, GSE32863, GSE43458, and GSE75037 datasets were randomly divided into a training cohort (70%, *N* = 516) and a test cohort (30%, *N* = 221). The “caret” package facilitated robust parameter tuning through grid search and cross‐validation techniques to identify optimal model parameter combinations and accurately estimate model performance. Residual distributions were analyzed and visualized using the “DALEX” package to assess feature importance in the four machine learning models. Performance evaluation and visualization of the four models were conducted using the “pROC” software package. By comparing area under the curve (AUC) values obtained through five‐fold cross‐validation among these models, we can determine the optimal choice based on the highest AUC.

### 2.8 Internal and External Dataset Validation Analysis for Diagnostic Performance of Plasma Proteins

The optimal machine learning model was selected to predict the diagnostic performance of the identified five plasma proteins. ROC curve analysis was independently conducted on five datasets (GSE30219, GSE30210, GSE32863, GSE43458, and GSE75037) to validate the diagnostic value of the model. Additionally, an external dataset (GSE40419) was employed for further validation of the predictive ability of the model. Higher AUC values indicate superior diagnostic performance of the identified plasma proteins.

### 2.9 Development and Evaluation of a LMRGS Clinicopathologic Nomogram

Utilizing the “rms” R package, we developed a nomogram model to individualize risk assessment and prognosis prediction for patients with LUAD, thereby optimizing treatment strategies. Each predictive variable was assigned a score based on its respective model weight or coefficient, reflecting its influence on the target variable. The cumulative score, known as the “total score,” represents the combined impact of all predictor variables. Additionally, deviation correction analyses were employed to evaluate LUAD risk using identified diagnostic markers in selected machine learning models. Furthermore, decision curve analysis (DCA) and sensitivity analysis were conducted to explore these predictive performances and enable the utilization of diagnostic markers for predicting treatment outcomes in patients.

### 2.10 RNA Isolation and Quantitative RT‐PCR Assay

Total RNA was isolated from LUAD tissues using the SPARKeasy Improved Tissue/Cell RNA Kit (SparkJade, AC0202). The complementary DNA (cDNA) was synthesized as per the manufacturer's instructions, utilizing the RevertAid First Strand cDNA Synthesis Kit (Thermo Fisher Scientific). qRT‐PCR was performed with the SYBR Green PCR kit (Takara Bio, Otsu, Japan) on a Step One Real‐Time PCR system (Thermo Fisher Scientific). The relative gene expression levels were quantified by employing the 2‐△△CT method. Primer sequences:

- *TNFRSF21*‐F ATTGGCACATACCGCCATGTT
- *TNFRSF21*‐R GGCTTGTGTTGGTACAATGCTC
- *BCHE*‐F GTCAGAGGGATGAACTTGACAG
- *BCHE*‐R TGAATCGAAGTCTACCAAGAGGT
- *FABP4*‐F ACTGGGCCAGGAATTTGACG
- *FABP4*‐R CTCGTGGAAGTGACGCCTT
- *LPL*‐F TCATTCCCGGAGTAGCAGAGT
- *LPL*‐R GGCCACAAGTTTTGGCACC
- *PLBD1*‐F CAAGATAAGTGGACCCGGAAAA
- *PLBD1*‐R TGTGCCATCACATAGCCTGTA

## 3 Results

### 3.1 Identification of Robust DE‐LMRGs for LUAD

**FIGURE 2:**
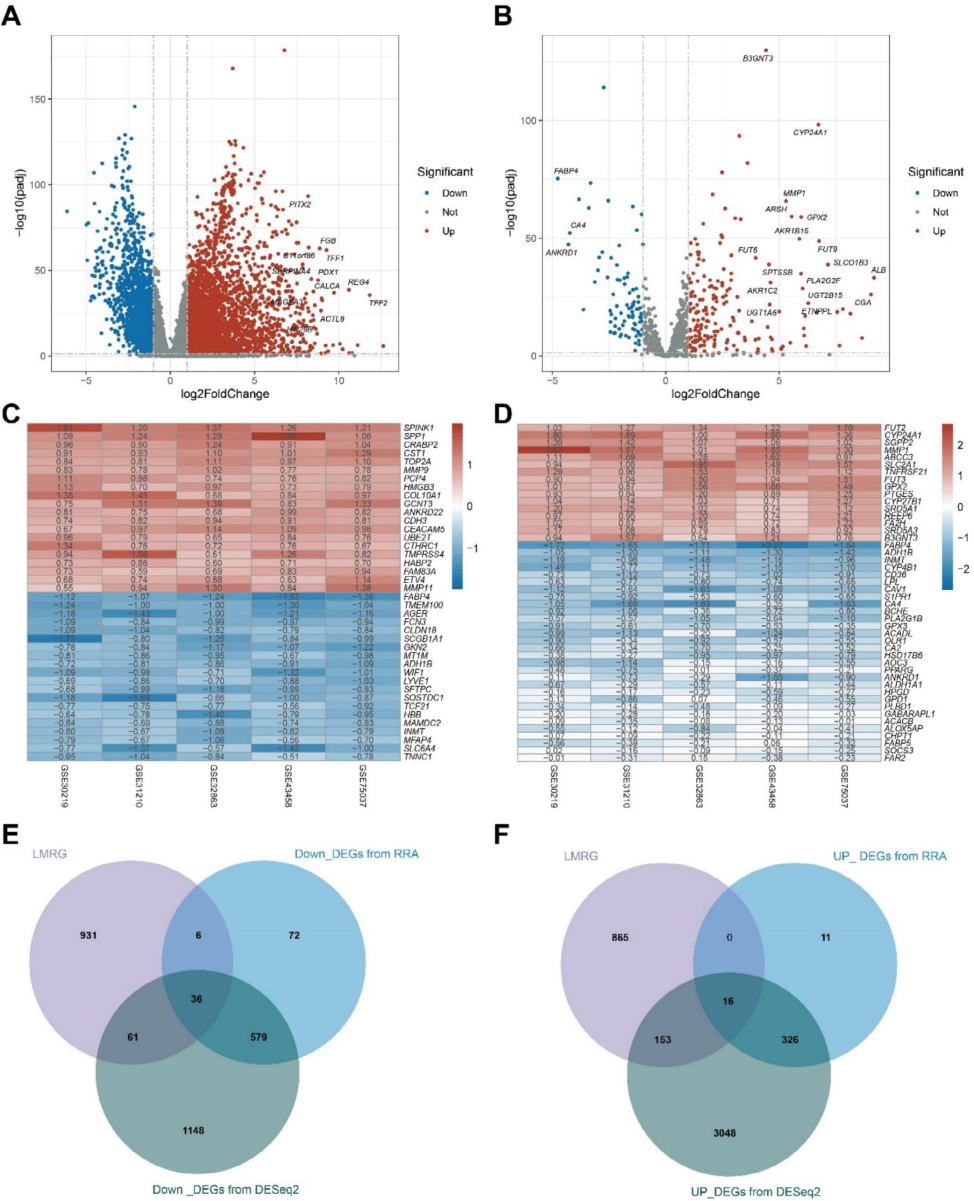
Identification of Robust DE‐LMRGs for LUAD. (A) Volcano plot of differentially expressed genes in TCGA‐LUAD dataset by DEseq2 analysis. (B) Volcano plot of differentially expressed LMRGs in TCGA‐LUAD dataset by DEseq2 analysis. (C) Heatmap of differentially expressed genes in GSE30219, GSE30210, GSE32863, GSE43458, and GSE75037 GEO dataset by RRA analysis. (D) Heatmap of differentially expressed LMRGs in GSE30219, GSE30210, GSE32863, GSE43458, and GSE75037 GEO dataset by RRA analysis. (E) Venn diagram for identifing down‐regulated DE‐LMRGs. (F) Venn diagram for identifing up‐regulated DE‐LMRGs.

To identify differentially expressed LMRGs (DE‐LMRGs) between LUAD and normal tissues, we initially extracted 1034 LMRGs from GSEA, KEGG, and REACTOME datasets (Figure S1; Table S1‐2). Subsequently, DESeq2 analysis identified 3543 upregulated genes and 1824 downregulated genes (Figure 2A; Table S3), including 185 upregulated LMRGs and 84 downregulated LMRGs (Figure 2B; Table S4), between 541 LUAD cases and 59 normal cases from TCGA database. Furthermore, RRA analysis revealed 353 upregulated genes and 693 downregulated genes (Figure 2C; Table S5), including 18 upregulated LMRGs and 42 downregulated LMRGs (Figure 2D; Table S6), between 532 LUAD tissues and 205 normal tissues in five integrated GEO datasets. Finally, a total of 16 upregulated genes and 36 downregulated genes were identified through the intersection of LMRGs, DEGs form TCGA and GEO datasets (Figure 2E,F; Table S7).

### 3.2 Identification of Potential Diagnostic Biomarkers and Therapeutic Targets From LMRGs for LUAD

**FIGURE 3:**
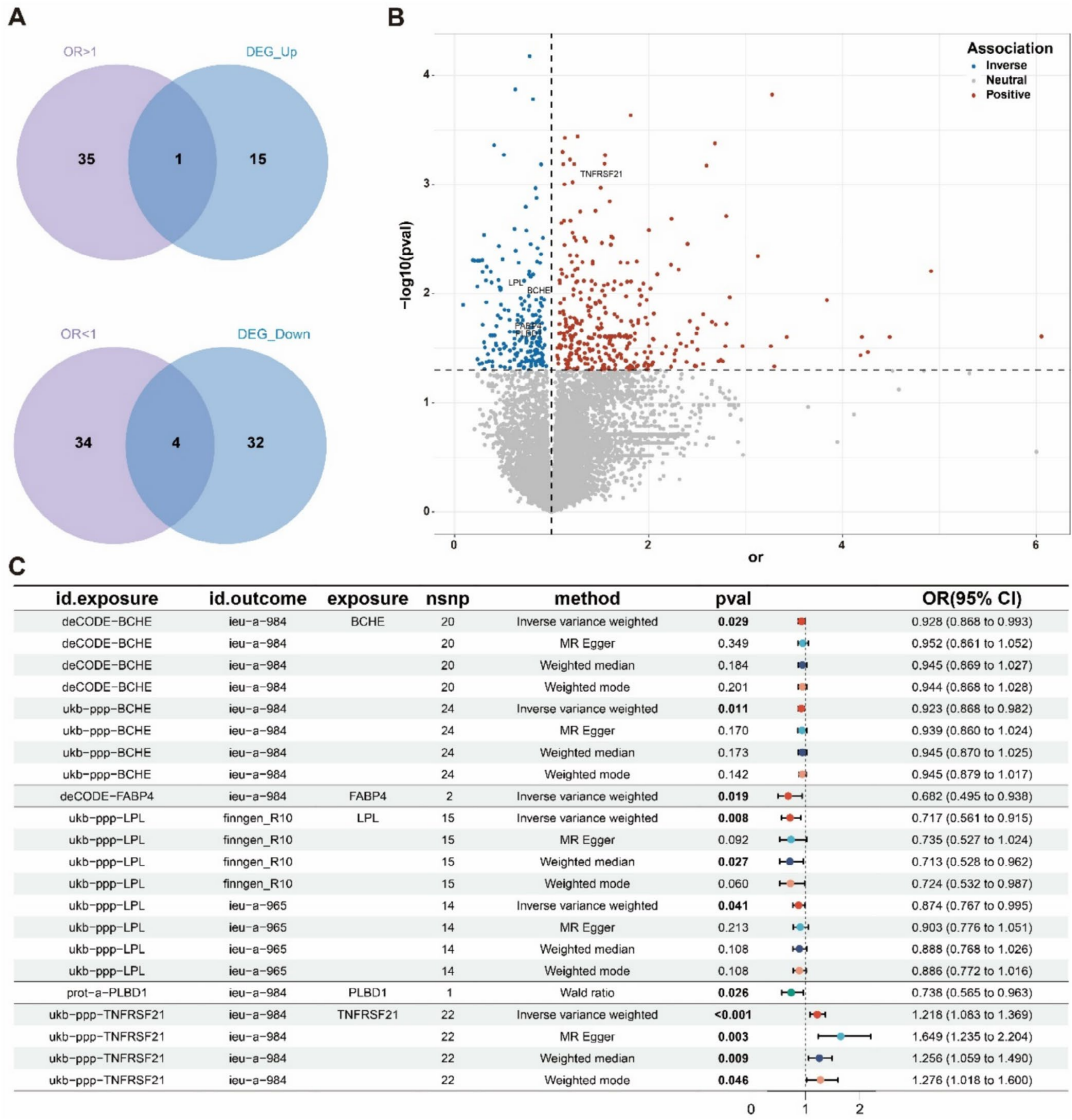
Identification of potential diagnostic biomarkers and therapeutic targets from LMRGs for LUAD. (A) Venn diagram illustrating the potential drug targets of LUAD between DE‐LMRGs and the MR‐identified proteins. (B) Volcano plots revealing the association of proteins based on OR in the deCODE and UKB‐PPP studies. (C) OR, 95% confidence intervals (CI), and *p* value for the effect of plasma proteins on LUAD estimated by the IVW, MR Egger, weighted median, weighted mode, or Wald ratio approaches.

Given their genetic susceptibility of individuals to diseases, plasma proteins expression quantitative trait loci (pQTLs) have emerged as promising targets for drug development and diagnostic biomarkers [30]. Utilizing LMRGs and screening criteria for IVs, we identified 189 pQTLs (UKB‐PPP), 129 pQTLs (prot‐a), and 327 pQTLs (deCODE) associated with LMRGs (Tables [Link], [Link]). MR analysis between these pQTLs and three outcomes of LUAD (ieu‐a‐965, ieu‐a‐984, and Finngen R10) revealed a total of 74 significant union‐proteins across the UKB‐PPP study (24 proteins), prot‐a (17 proteins), and deCODE study (48 proteins) with a statistical significance threshold of *p* value < 0.05 (Table S11). The Venn diagram and volcano plot demonstrated that one risk‐associated LMRG gene *TNFRSF21*, along with four protective LMRG genes *BCHE*, *FABP4*, *LPL*, and *PLBD1*, were implicated in LUAD development (Figure 3A,B). MR analysis using the IVW method showed a significant positive causal association between TNFRSF21 and LUAD, with an odds ratio (OR) of OR = 1.218 (95% CI: 1.083–1.369) and *p* < 0.049 (Figure 3C). BCHE, FABP4, LPL, and PLBD1 exhibited significant negative associations with LUAD accompanied by *p* < 0.05 (Figure 3C). The OR values for FABP4 and PLBD1 were 0.682 (95% CI: 0.495–0.938) and 0.738 (95% CI: 0.565–0.963), respectively. The OR values for BCHE in both deCODE and UKB‐PPP databases were found to be significantly associated with LUAD, with ORs of 0.928 (95% CI: 0.868–0.993) and 0·923 (95% CI: 0.868–0.982), respectively. Additionally, the OR value for LPL in the UKB‐PPP database was observed as significantly associated with LUAD in both Finngen R10 dataset and ieu‐a‐965 dataset, yielding ORs of 0.717 (95% CI: 0.561–0.915) and 0.874 (95% CI: 0.767–0.995), respectively. These results suggest that BCHE, FABP4, LPL, PLBD1, and TNFRSF21 were potential diagnostic biomarkers and therapeutic targets for LUAD.

### 3.3 Construction of Machine Learning Models

**FIGURE 4:**
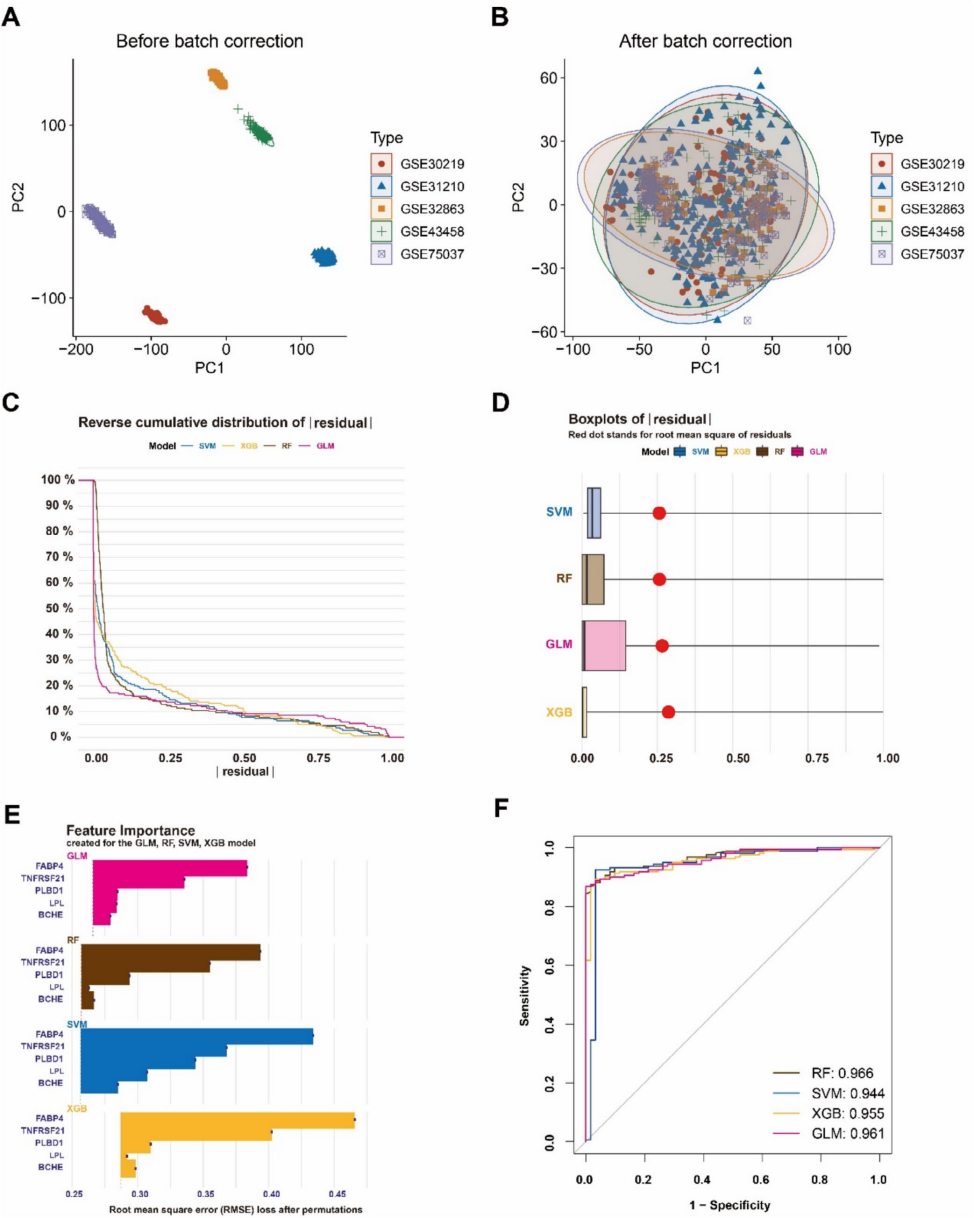
Screening machine learning models for diagnostic markers of LUAD. (A) PCA analysis for five GEO datasets before batch correction. (B) PCA analysis for five GEO datasets after batch correction. (C) Cumulative residual distribution for SVM, RF, GLM, and XGB machine learning models. (D) Residuals boxplots for each machine learning model. (E) The important features of five plasma proteins in each machine learning model. (F) ROC curves for four machine learning models using five‐fold cross‐validation on the test cohort.

Since the persistent suitability of proteins as diagnostic markers, we employed four machine learning models (RF, SVM, GLM, and XGB) to evaluate the diagnostic performance of the aforementioned candidate genes. Firstly, in order to obtain a large sample dataset for accurate machine learning analysis, we standardized and merged the expression values of each gene from GSE32863, GSE30219, GSE31210, GSE43458, and GSE75037 datasets to create a comprehensive sample pool consisting of 532 LUAD samples and 205 normal tissues. Principal component analysis (PCA) also demonstrated successful elimination of batch effects across these five datasets (Figure 4A,B). Subsequently, the “DALEX” package was utilized to visualize test set residuals across each model. Notably, RF machine learning models exhibited relatively lower residuals (Figure 4C,D). The five plasma proteins in each model were ranked using root mean square error (RMSE), with FABP4 displaying the highest feature importance followed by TNFRSF21, PLBD1, LPL, and BCHE (Figure 4E). Finally, we evaluated the performance of each algorithm on the large dataset using five‐fold cross‐validation and calculated the AUC of the ROC curve. The RF machine learning model demonstrated the highest AUC (AUC‐RF = 0.966, AUC‐SVM = 0.944, AUC‐XGB = 0.955, AUC‐GLM = 0.961; Figure 4F).

### 3.4 Evaluation of the Prediction Model

**FIGURE 5:**
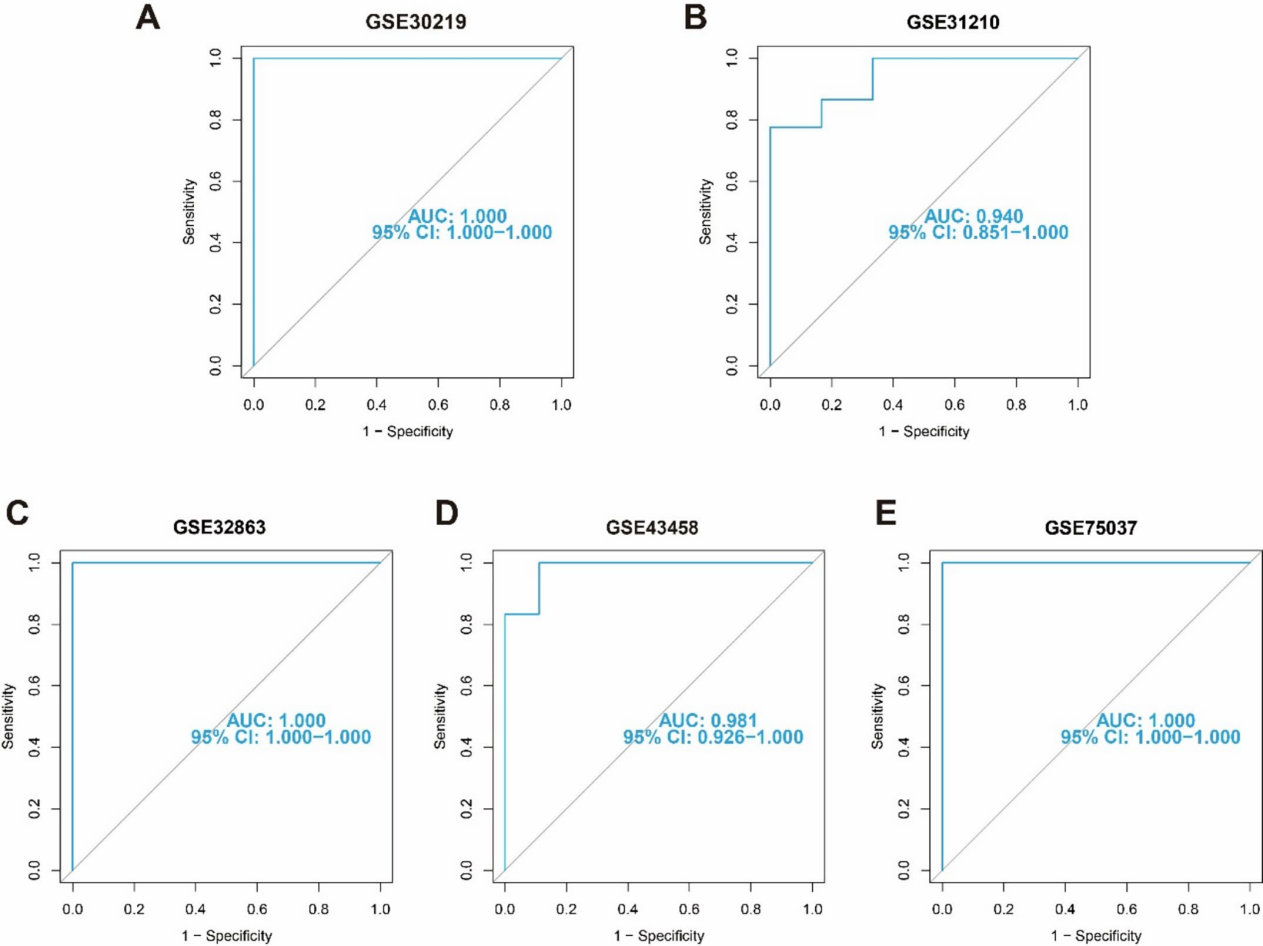
ROC curve of the four plasma protein‐based RF model. ROC curve of the four plasma proteins‐based RF model in GSE30219 (A), GSE31210 (B), GSE32863 (C), GSE43458 (D), and GSE75037 (E) cohorts.

To further assess the predictive efficacy of the RF model, we validated our five‐gene prediction model across five independent datasets. The ROC curve analysis demonstrates that the RF predictive model based on the five‐gene signature exhibits commendable performance. Specifically, the AUC values for datasets GSE30219, GSE30210, GSE32863, GSE43458, and GSE75037 are 1.0, 0.940, 1.0, 0.981, and 1.0, respectively (Figure 5A–E). These findings underscore the robustness of our predictive model.

**FIGURE 6:**
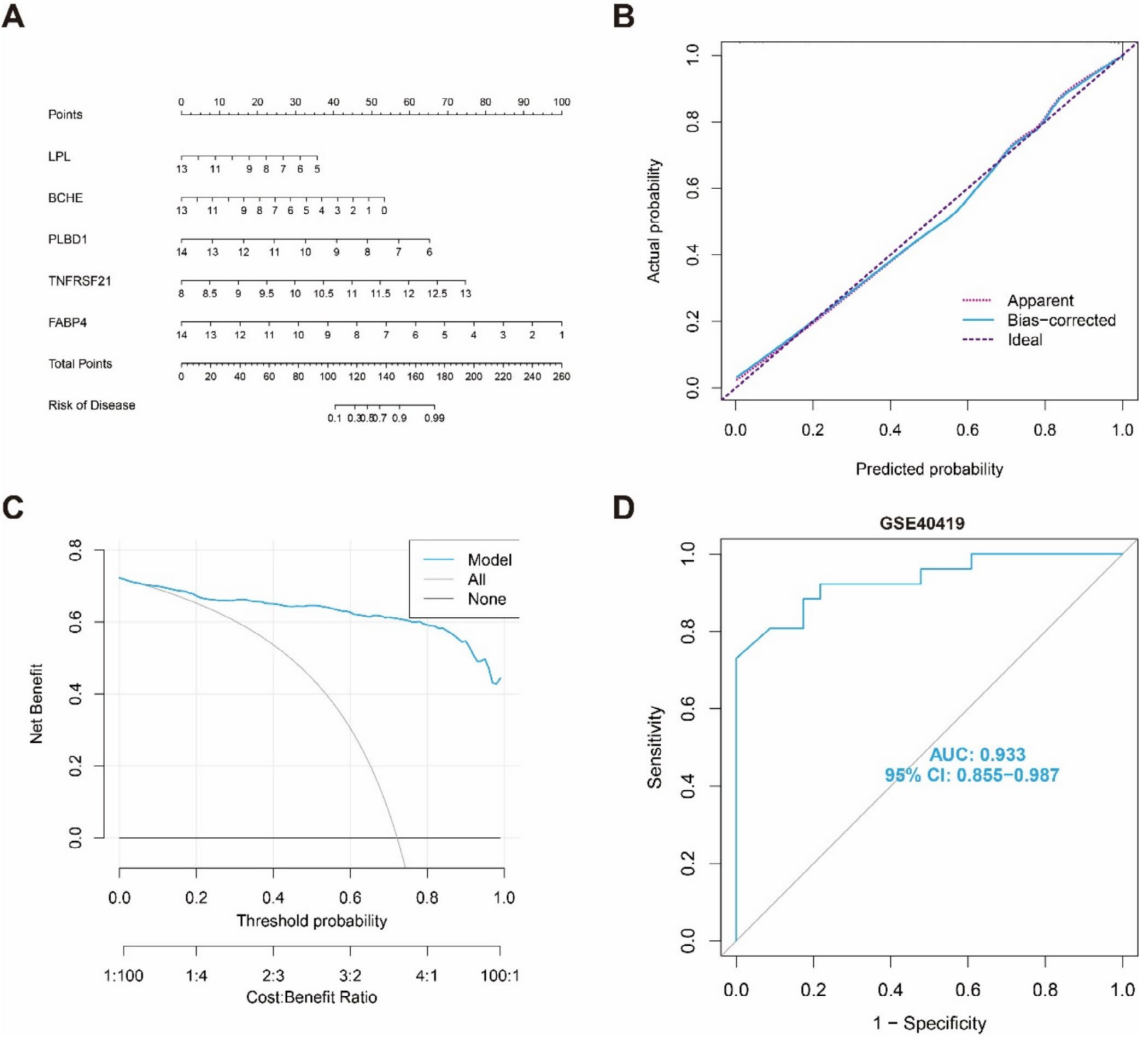
Validation of five diagnostic biomarkers. (A) Nomogram of the five plasma proteins for predicting the risk of LUAD. (B, C) Calibration curve (B) and DCA (C) to evaluate the nomogram's predictive efficiency. (D) ROC analysis verifying the five proteins‐based RF model in external validation cohorts (GSE40419).

Moreover, a nomogram was employed to estimate the scores of FABP4, TNFRSF21, PLBD1, LPL, and BCHE in order to facilitate intuitive prediction of the risk of onset in a specific patient population (Figure 6A). The calibration curve demonstrates that the RF model exhibits high predictive accuracy with minimal discrepancy between the actual and predicted risks associated with LUAD patients (Figure 6B). Furthermore, DCA analysis reveals that our nomogram possesses exceptional accuracy and has potential utility in guiding clinical decision‐making (Figure 6C). Finally, we validated our five‐gene prediction model using an external validation dataset and observed excellent performance of the five‐gene signature with an AUC value of 0.933 in the GSE40419 dataset (Figure 6D), indicating that our diagnostic biomarkers are equally effective at distinguishing LUAD patients from normal individuals.

### 3.5 The Experiment of Genes Involved in the Risk Signature

**FIGURE 7:**
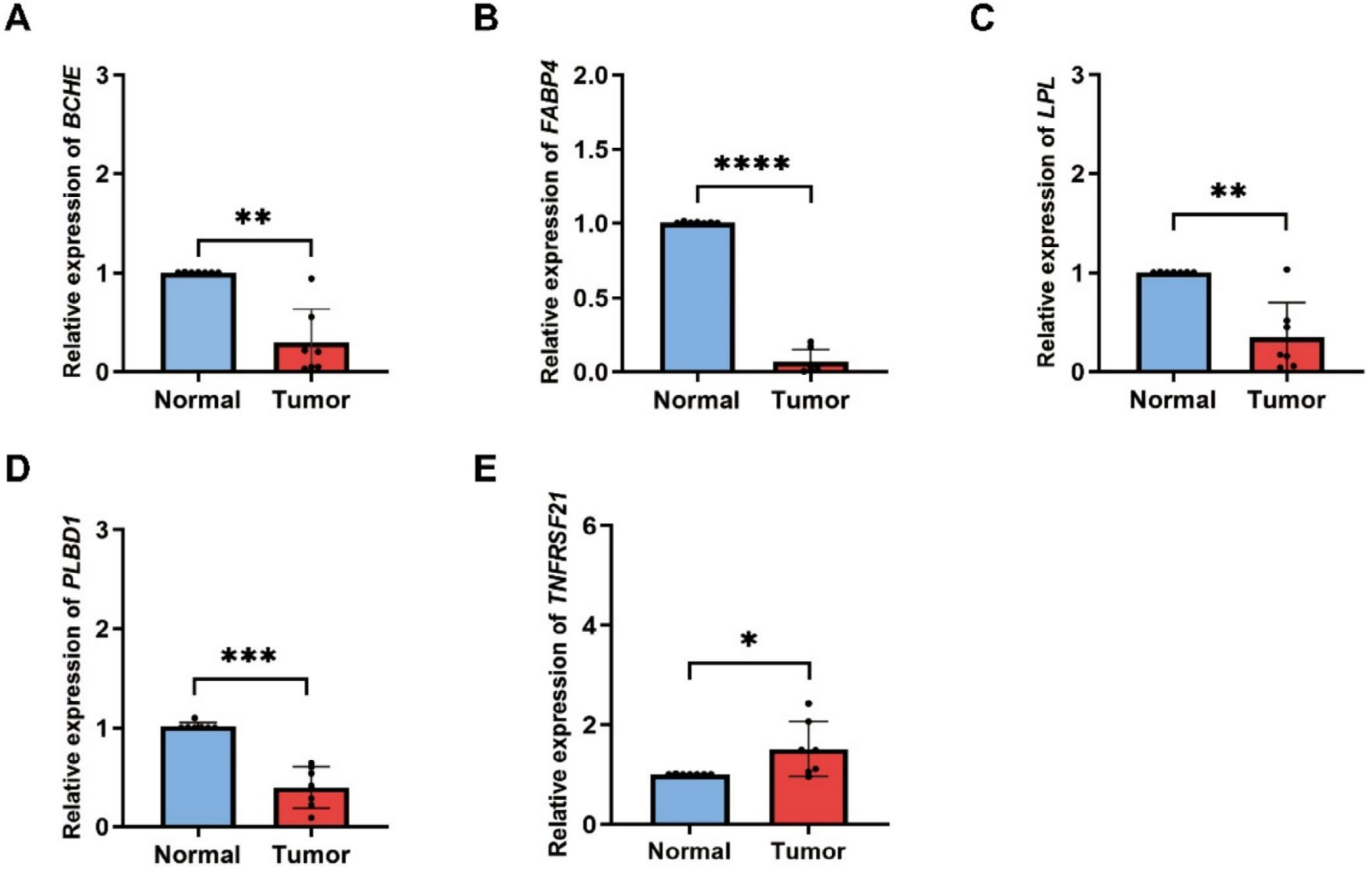
The experimental validation of hubgenes. The expression of *BCHE* (A), *FABP4* (B), *LPL* (C), *PLBD1* (D) and *TNFRSF21* (E) in normal tissue and tumor tissue of LUAD patients. A *t*‐test was used to compare the expression of genes between normal and tumor. *p < 0.05, **p < 0.01, ***p < 0.001, ****p < 0.0001.

In order to validate the reliability of our findings, we conducted additional biological experiments. For this study, we acquired seven pairs of samples comprising both normal and tumor tissues from patients diagnosed with LUAD. Remarkably, these samples exhibited consistent expression patterns. As anticipated, the expression levels of *BCHE*, *FABP4*, *LPL*, and *PLBD1* were found to be significantly downregulated in all seven pairs of LUAD tissue samples (Figure 7A–D). Furthermore, *TNFRSF21* demonstrated a notable upregulation across all seven pairs of LUAD tissue samples (Figure 7E).

## 4 Discussion

Effective diagnostic biomarkers and drug targets are crucial for the clinical diagnosis and treatment strategy of LUAD [31, 32]. Disordered lipid metabolism is a malignant characteristic of LUAD that contributes to its carcinogenesis and progression [12, 33]. In this study, we identified 66 upregulated LMRGs and 36 downregulated LMRGs from TCGA and GEO datasets using DESeq2 and RRA analysis after selecting 1034 LMRGs from GSEA, KEGG, and REACTOME datasets. We then estimated the causal relationship between plasma proteins and LUAD in ieu‐a‐965, ieu‐a‐984, and Finngen R10 cohort by analyzing pQTLs of these102 LMRGs in deCODE, prot‐a, UKB‐PPP Study through MR analysis. BCHE, FABP4, LPL, and PLBD1 were significantly negatively associated with LUAD accompanied by OR > 1 and *p* < 0.05 while TNFRSF21 was significantly positively causally associated with LUAD with OR < 1 and *p* < 0.049. These five plasma proteins were considered as potential therapeutic targets for treating LUAD. Finally, the exceptional diagnostic performance of these five LMRGs was demonstrated through machine learning and validation using internal or external datasets, with AUC values ranging from 0.933 to 1.000. In conclusion, our findings highlight BCHE, FABP4, LPL, PLBD1, and TNFRSF21 as novel and reliable diagnostic markers and potential therapeutic targets for LUAD.

As a member of the cholinesterase family, butyrylcholinesterase (BCHE) is an enzyme that catalyzes the hydrolysis of acylcholine. It belongs to the alpha‐glycoprotein family and is present in both the nervous system and liver [34]. BCHE has been implicated in tumor cell choline metabolism, potentially affecting their energy supply and signal transduction. Previous studies have demonstrated associations between BCHE expression levels and cell adhesion, differentiation, apoptosis, as well as tumorigenesis [35, 36]. Furthermore, BCHE expression levels may vary according to tumor stage [37]. Notably, high expression of BCHE has been reported in breast cancer [36], while minimal expression has been observed in colorectal cancer [38]. In this study, we observed low levels of BCHE expression specifically in LUAD.

Fatty acids in adipose tissue (FABP4), a member of the fatty acid‐binding protein superfamily [39], play a pivotal role in regulating intracellular transport and metabolism of fatty acids. This regulation is crucial for lipid metabolism and signal transduction, thereby governing cell growth, differentiation, and immune response. Notably, FABP4 exhibits low expression levels in endometrial cancer [40]. Furthermore, the expression of FABP4 regulates the development of obesity‐associated breast cancer and pancreatic cancer [41, 42]. Our study revealed that FABP4 was a protective factor for LUAD patients.

Low expressions of lipoprotein lipase (LPL) have been correlated with worse overall survival in LUAD patients [12, 43]. This association may be attributed to the involvement of LPL in lipid metabolism, thereby contributing to the maintenance of tumor microenvironment homeostasis and suppression of tumor growth and metastasis. Our study corroborates these findings through the utilization of MR and machine learning analysis.

TNFRSF21, also known as Death Receptor 6 (DR6) [44], belongs to the tumor necrosis factor receptor superfamily [45]. It exhibits significant upregulation in certain lung cancer cell lines and may be associated with cancer progression, invasion, and metastasis [46]. TNFRSF21 plays a role in apoptosis induction, immune system regulation, and inhibiting immune evasion and metastasis in lung cancer. In our research, we observed elevated expression of TNFRSF21 in LUAD and identified it as a potential risk factor.

The phospholipase B domain containing 1 (PLBD1) was initially identified in neutrophils [47]. It was predicted to possess phospholipase activity and to be involved in the catabolic process of phospholipids. Vanhaverbeke et al. discovered that PLBD1 and QSOX1 were upregulated, serving as novel independent markers for left ventricular dysfunction following myocardial infarction [48]. In contrast to our findings, PLBD1 was downregulated and considered a protective factor for LUAD. Furthermore, we identified that together with BCHE, FABP4, LPL, and TNFRSF21, it could serve as promising diagnostic markers for LUAD.

Cao et al. identified a panel of 11 LMRGs, namely *LDHA*, *SEC14L3*, *LPL*, *ACSS3*, *GAPDH*, ESYT3, *SEC14L4*, *CYP17A1*, *CFTR*, *SLC16A1*, and *CIDEC*, that exhibited high accuracy in predicting overall survival (OS) for LUADs. The constructed risk model using LASSO regression yielded an AUC of 0.722 for 5‐year OS [49]. Similarly, another study demonstrated the prognostic value of a nine‐gene LMRGs signature consisting of *CYP4B1*, *KLF4*, *DPEP2*, *PTGDS*, *CYP27A1*, *ACSS3*, *HSD17B13*, *HPGDS*, and *FA2H* with an AUC of 0.646 for 5‐year OS based on time‐dependent ROC curves [17]. Furthermore, the emergence of plasma proteins as highly promising diagnostic markers due to their minimally invasive nature and ease of detection is widely acknowledged. By integrating MR analysis with DEGs and machine learning techniques in our study, five plasma proteins were identified as potential therapeutic targets and diagnostic markers with excellent performance in internal and external validation databases (AUC values ranging from 0.933 to 1.000). Compared with previous results, the five plasma protein signature we proposed demonstrates superior diagnostic accuracy for LUAD.

## 5 Conclusion

Overall, we have identified five biomarkers associated with LMRGs as potential drug targets for LUAD. These five proteins exhibited exceptional diagnostic performance, with AUC values ranging from 0.933 to 1.000. Therefore, BCHE, FABP4, LPL, PLBD1, and TNFRSF21 represent novel and reliable diagnostic markers and therapeutic targets for LUAD that hold promise for effective clinical applications.

## Author Contributions

S.W. and Z.Z. were involved in the conception and design of the study; S.W., Z.G., and T.X. completed the data analysis; S.W. and G.F. performed the biological validation experiment; S.W. finished writing the manuscript; T.X. and Z.L. contributed to manuscript revisions. Z.Z. supervised the overall study. All authors reviewed and approved the final manuscript.

## Ethics Statement

The studies involving human participants were reviewed and approved by the Tianjin Medical University Cancer Institute and Hospital Ethical Board, which gave the study its approval. Batch number: bc2023152.

## Conflicts of Interest

The authors declare no conflicts of interest.

## Supporting information


**Figure S1.** LMRGs from GSEA.

Table S1‐1.

Table S1‐2.

Table S2.

Table S3.

Table S4.

Table S5.

Table S6.

Table S7.

Table S8.

Table S9.

Table S10.

Table S11.

## Data Availability

The datasets supporting the conclusions of this article are available on the GEO website (https://www.ncbi.nlm.nih.gov/geo/) and the Cancer Genome Atlas (TCGA) database (https://portal.gdc.cancer.gov/). The original contributions presented in the study are included in the article and Supporting Information.
