# Supplementary figures and images for: Identifying Lipid Metabolism‐Related Therapeutic Targets and Diagnostic Markers for Lung Adenocarcinoma by Mendelian Randomization and Machine Learning Analysis
Source: Thorac Cancer. 2025 Mar 19;16(6):e70020. doi: 10.1111/1759-7714.70020 (PMC11922676; doi:10.1111/1759-7714.70020)

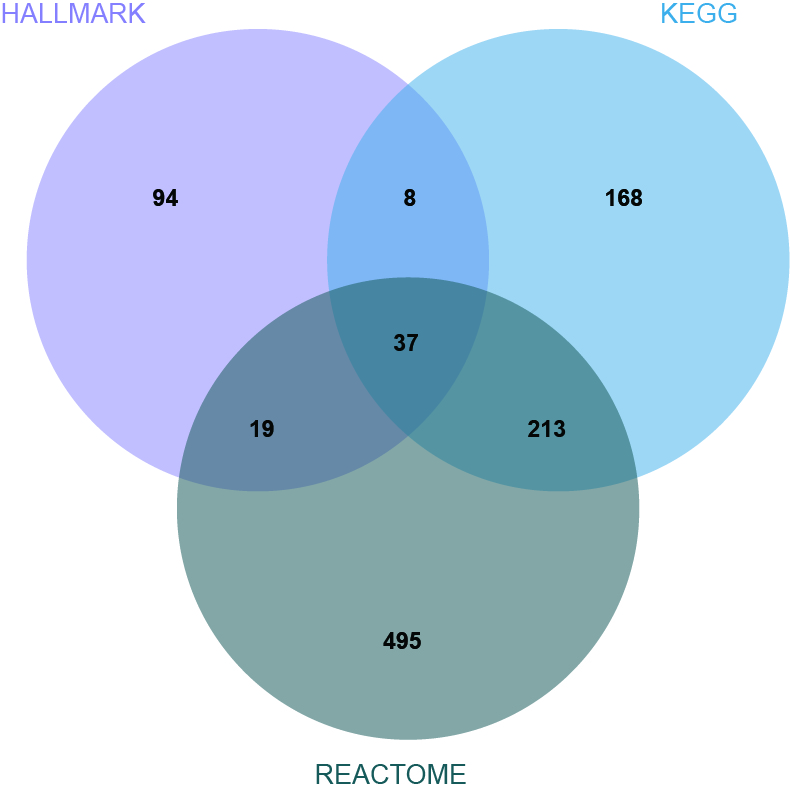

Supplement: Supplementary file 1 — Figure S1. LMRGs from GSEA. [file TCA-16-e70020-s013.jpg]
